# Multi-Contrast Imaging and Digital Refocusing on a Mobile Microscope with a Domed LED Array

**DOI:** 10.1371/journal.pone.0124938

**Published:** 2015-05-13

**Authors:** Zachary F. Phillips, Michael V. D'Ambrosio, Lei Tian, Jared J. Rulison, Hurshal S. Patel, Nitin Sadras, Aditya V. Gande, Neil A. Switz, Daniel A. Fletcher, Laura Waller

**Affiliations:** 1 Graduate Group in Applied Science and Technology, University of California, Berkeley, CA 94720, USA; 2 Department of Electrical Engineering and Computer Sciences, University of California, Berkeley, CA 94720, USA; 3 Department of Bioengineering, University of California, Berkeley, CA 94720, USA; Glasgow University, UNITED KINGDOM

## Abstract

We demonstrate the design and application of an add-on device for improving the diagnostic and research capabilities of CellScope—a low-cost, smartphone-based point-of-care microscope. We replace the single LED illumination of the original CellScope with a programmable domed LED array. By leveraging recent advances in computational illumination, this new device enables simultaneous multi-contrast imaging with brightfield, darkfield, and phase imaging modes. Further, we scan through illumination angles to capture lightfield datasets, which can be used to recover 3D intensity and phase images without any hardware changes. This digital refocusing procedure can be used for either 3D imaging or software-only focus correction, reducing the need for precise mechanical focusing during field experiments. All acquisition and processing is performed on the mobile phone and controlled through a smartphone application, making the computational microscope compact and portable. Using multiple samples and different objective magnifications, we demonstrate that the performance of our device is comparable to that of a commercial microscope. This unique device platform extends the field imaging capabilities of CellScope, opening up new clinical and research possibilities.

## Introduction

Optical microscopy is an important tool for disease screening and diagnosis throughout the world; however, access is often restricted to centralized hospitals due to the cost and complexity of imaging hardware. Significant resources have been devoted to developing portable and affordable compact microscopes for remote clinical applications [1–12]. Compact microscopes based on mobile phones, including CellScope [13, 14], have demonstrated that microscopy can be effectively performed outside of hospitals and diagnostic laboratories by minimally trained healthcare workers, that images can be transmitted for confirmation of diagnosis, and that phone-based computational analysis can be used to provide automated diagnosis. These mobile microscopes complement a host of other new devices for health monitoring on smart phones [15–18]. Here, we demonstrate a new variation of the CellScope microscope which incorporates recently developed techniques of computational illumination [19–21] to enable new imaging modalities, including darkfield, phase imaging and digital refocusing.

Contrast in clinical microscopy is usually obtained through chemical staining or tagging to enhance specific features of a sample, requiring extensive sample preparation. Label-free contrast methods which do not require staining (e.g. darkfield, phase contrast, DIC) have not yet found widespread use as a field diagnostic tool [10], in part due to significant expense and complexity of the related optical hardware. We implement here a simple phase imaging modality, Differential Phase Contrast (DPC) [22–24], which requires only two images with complementary illumination patterns. The result is qualitatively similar to differential interference contrast (DIC) imaging, but has the advantage of being quantitative, which enables measurement of cell volume and dry mass [25, 26], as well as cell confluence and tracking studies [27]. Since brightfield, darkfield and phase imaging are achieved in our system simply by switching the illumination pattern, we are able to display of all three contrast modes simultaneously by synchronizing the LED illumination patterns with the camera acquisition [21]. Image capture is quasi-real-time, with a phone-processor-limited frame rate of 0.43 Hz. Given trends in the industry toward ever-more processing power, real-time (5–8Hz) imaging is reasonable to expect within a couple of product cycles.

Using the same hardware, our Computational CellScope also implements lightfield digital refocusing, so that a sample focus can be changed after the fact (without mechanically changing focus) and 3D image stacks can be extracted for both intensity and phase modes. Further, constant focus correction (auto-focusing) can be implemented in post-processing for long time-lapse studies. The digital refocusing is achieved by sequentially illuminating the sample from each of the LEDs that lie inside the numerical aperture (NA) of the objective, then post-processing to form a stack of through-focus images of intensity [19, 28] or phase contrast [20]. For thick samples, the result also provides a 3D reconstruction of the sample, similar to limited angle tomography.

The computational illumination techniques used here have been previously demonstrated in a traditional microscope using a planar LED array [19–21, 29]. The purpose of the LED array is to flexibly pattern illumination angles at the sample by turning on different sets of LEDs corresponding to different illumination angles. The optimal arrangement of LEDs, however, is not planar but rather a dome shape [30], which we utilize here. The domed arrangement provides significant improvements in intensity uniformity and light throughput, since LEDs can be directionally biased and arranged at uniform radius from the sample. These benefits contribute to increased signal-to-noise ratio (SNR) in the darkfield images, allowing effective high angle illumination patterning and shorter exposure times.

The flexibility and speed of the programmable LED array illuminator, as well as the lack of moving parts and low cost, make the hardware very amenable to modification as a CellScope attachment. In order for our device to be practically useful in the field, we have here enforced the requirement that all of our processing and control be performed on the smartphone, without use of a PC. Thus, the device can be field-deployable as a simple add-on to CellScope. In the following sections we detail the design and performance of the hardware and software of our new Computational CellScope device.

## Results

### Hardware

**Fig 1:**
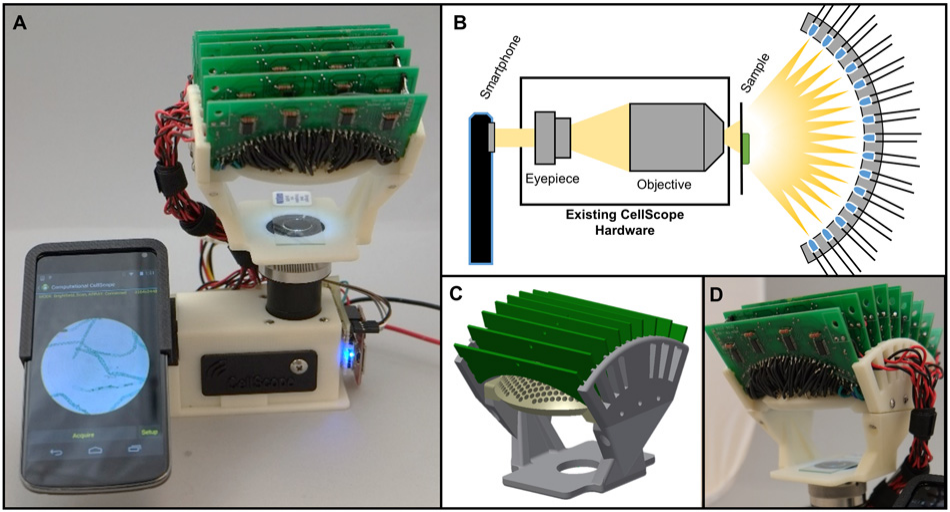
Computational CellScope. **A.** Device observing a sample using a Nexus 4 smartphone. **B.** Optical schematic of the CellScope device with our custom-made domed LED illuminator. **C.** CAD assembly of the dome. **D.** Assembled dome and control circuitry.

**Fig 2:**
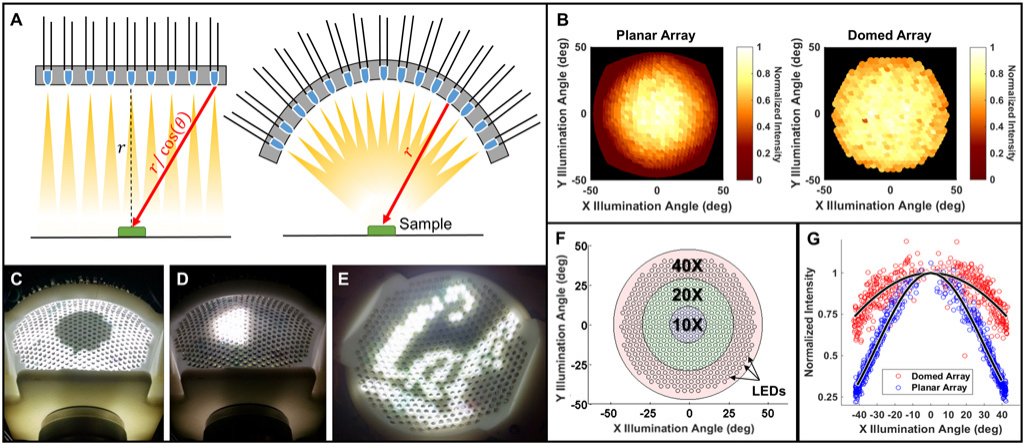
Domed LED Illuminator. **A.** Visual comparison of a planar LED array with a domed array. Since the intensity of a spherical wave drops as a function of the inverse square of radius, the illumination at the sample depends on the distance between the LEDs and the sample. In the planar case (left), LED distance *r* increases as a function of illumination angle, causing weaker illumination at higher angles. A domed LED array (right) eliminates this variation (*r* is constant). **B.** Normalized mean pixel intensities measured at the sensor for the planar and domed arrays. Intensity decreases as a function of angle in both cases, but much more strongly in the case of the planar geometry. Values were normalized to the central LED’s brightness in both cases. **C.** Illumination pattern used to acquire dark field images with a 0.25 NA objective. **D.** Illumination pattern used to synthesize differential phase contrast images with a 0.25 NA objective. **E.** Illustration of the arbitrary illumination patterning capabilities of the device. **F.** Plot illustrating the relative objective NA for several common magnifications, as compared to our dome’s LED placement (small black circles). **G.** Normalized measured intensity falloff as a function of angle relative to the optical axis for the domed and planar LED arrays. Falloff is proportional to cos *θ* for the domed geometry and ∼ cos^4^ *θ* for the planar geometry. Black lines are cos *θ* and cos^4^ *θ* fits for the domed and planar geometries, respectively. The domed geometry exhibits significant improvements in intensity at large angles of illumination.

The Computational CellScope hardware involves a custom-built domed LED illuminator attached to an inverted variant of the CellScope smartphone-based microscope platform (see Fig 1). The CellScope used here is a finite-conjugate transmission microscope coupled to an Android-based Nexus 5 smartphone (LG Electronics/Google) as described in in Skandarajah, et al. [14]. Our domed illuminator hardware is compatible with all smartphones and tablets that are used with the existing CellScope, including the iPhone 4S, 5, 5S, and 6 (Apple, Inc.), as well as several Android devices. Phones are mounted via modular 3D printed mounts adapted to each specific smartphone model. Hardware changes were entirely on the illumination side, where we have replaced the original single LED light with our domed illuminator consisting of 508 individually addressable broad spectrum (white) LEDs. Our domed LED arrangement was inspired by the opto-mechanical geometry of the AWARE gigapixel camera [31]. LEDs are uniformly distributed in an (approximately) hexagonal packing pattern across a 77 degree cone of angles corresponding to an illumination NA of 0.62. Thus, darkfield imaging is feasible for objectives with NA smaller than 0.62 (as illustrated in Fig 2F), and both phase and digital refocusing are possible for all objective NAs. The dome assembly was is secured to a custom stage that attached to the top of the CellScope objective; the stage and circuit board holders were 3D printed using low-cost ABS plastic. In general, the design is modular and features simple electronics, including the use of the inexpensive and widely used Arduino micro-controller platform. Phone mounts can be swapped out for upgrading to new models and objectives can be replaced for varying the magnification of the system. While our addition involves custom LED drive circuitry and a 3D printed structure, complexity was kept low to preserve the low-cost nature of CellScope. Part counts, cost and especially size may be further reduced in design-for-manufacture. The size of the illuminator could be reduced to essentially the dimensions of the dome itself, and cost could be comparable to the price of a modern smartphone, matching and improving upon the functionality of a full-size microscope at a fraction of the cost.

The domed LED arrangement provides significantly better light efficiency than the planar LED arrays used in previous work, enabling shorter acquisition times and more efficient power use. These advantages could be crucial for mobile microscopy applications where power is a scarce resource, and shorter exposure times reduce motion blur artifacts due to unstable experimental conditions. The power benefits are a result of two phenomena, shown in Fig 2A. The first is that off-axis LEDs in a planar array will have a larger LED-to-sample distance and thus decreased intensity at the sample. For example, if we assume that each LED is a point emitter, the intensity falloff due to increased distance can be expressed as *I*(*θ*) = *I*_0_ cos^2^ *θ*, where *I*_0_ is the intensity at the sample from the on-axis LED and is illumination angle. The second improvement in light efficiency comes from the fact that LEDs have significant angular variation in intensity (typically emitting more light in the forward direction). In a planar array, the LEDs at higher angles provide less effective illumination, a problem corrected by the dome geometry, where all LEDs are radially oriented. In both the domed and planar geometries we note that intensity further decreases with a final factor of cos *θ* due to the smaller profile of objective window when viewed off-axis; combining these factors and assuming a Lambertian (∼ cos *θ*) angular dependence for physical (non-point-source) LEDs results in an expected intensity falloff of ∼ cos^4^ *θ* for the planar geometry but only ∼ cos *θ* for the domed geometry, a vast improvement at high incidence angles. Thus, the difference between geometries is proportional to *cos* ^3^ *θ*, or a factor of > 50% at 40° and 99% at 77° incidence, having a substantial impact on required exposure times. Such behavior matches well with our experimental measurements (Fig 2B–2G), where the measured intensity is shown for both geometries out to 40 incidence. Variations in intensity between LEDs may also come from electrical variations such as batch differences in controller chips and resistor tolerances.

#### Multi-Contrast Imaging

To achieve brightfield, darkfield and phase contrast simultaneously, we time-multiplex images taken with different LED patterns and post-process them on the smartphone to synthesize pseudo-real-time multi-contrast imaging, as in [21]. Brightfield images correspond to illumination by LEDs that lie within the cone of angles described by the objective numerical aperture (NA). Darkfield images are obtained by illuminating the sample from angles beyond the angular acceptance of the objective (Fig 2C) [19]. Since different objectives have different NA, one must specify in the software which objective is being used, with larger NA corresponding to a larger brightfield region of LEDs. Our dome is designed to enable darkfield contrast for any objective of NA < 0.62, roughly corresponding to a typical 40× objective.

**Fig 3:**
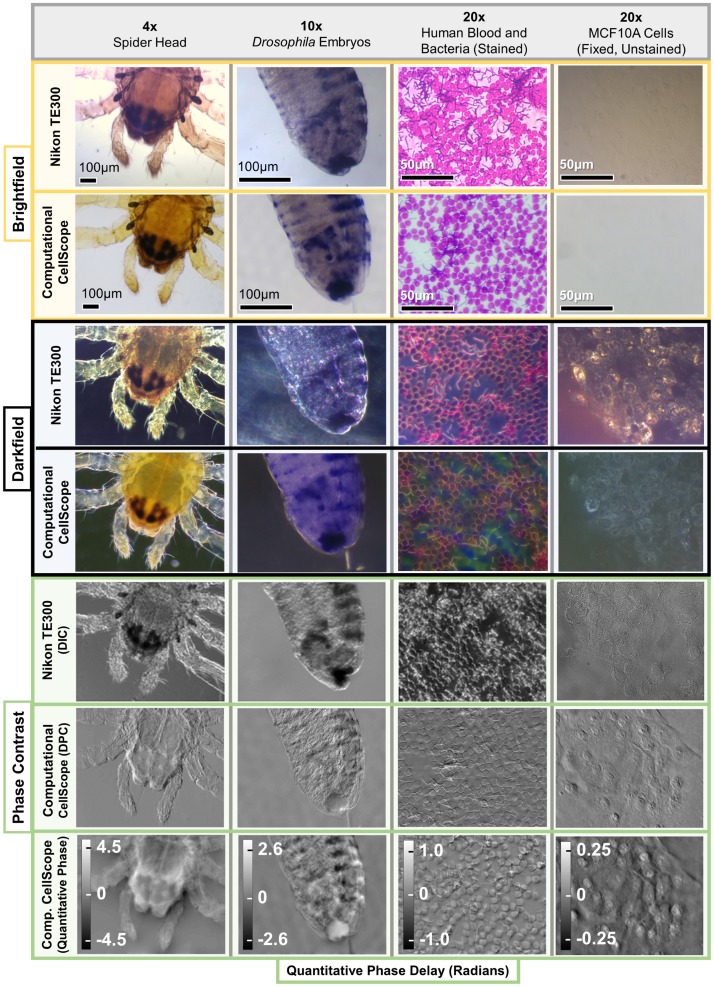
Image Results Compared to a Standard Microscope. Computational CellScope acquires brightfield and darkfield images of similar quality to a standard upright microscope (Nikon TE300) without the use of hardware inserts. Additionally, it enables phase imaging using Differential Phase Contrast (DPC), which contains similar information to standard phase contrast imaging, and can be inverted to obtain quantitative phase of the sample (bottom row). Differences in color shades are caused by the relative differences in hue of the halogen lamp and the white LEDs. Note the additional dark features in DIC results, as compared to DPC, illustrating mixing of phase and absorption information in DIC. In the rightmost column, we show images for an unstained transparent sample, illustrating the utility of phase imaging methods for label-free imaging.

Phase contrast can be achieved in a single-shot image by any asymmetric illumination pattern [32, 33]. Here, we choose to employ a differential phase contrast (DPC) scheme [20, 23, 24, 34], which requires two images having complementary illumination patterns, because it gives good phase contrast at all spatial frequencies and can be quantitatively interpreted. The method involves sequentially illuminating the sample with the two opposite halves of the brightfield circle while capturing an intensity image for each. For example, one may first take an image, *I*_R_, with only the right half of the LEDs on and then a second image, *I*_L_, with only the left half of LEDs on (see Fig 2D). The two images are processed as follows to obtain brightfield and phase contrast:

$$\begin{array}{c} I_{\text{BF}}=I_{\text{L}}+I_{\text{R}},\;I_{\text{DPC}}=\frac{I_{\text{L}}-I_{\text{R}}}{I_{\text{L}}+I_{\text{R}}}, \end{array}$$ (1)

where *I*_BF_ is the brightfield image and *I*_DPC_ is the phase contrast image. Since the LEDs are mutually incoherent, adding the two images gives an equivalent brightfield image and subtracting them produces phase contrast, due to asymmetric clipping in Fourier space. The intensity of the DPC image can be shown to be approximately proportional to the first derivative of phase along the direction of illumination asymmetry [34], and different axes of rotation can be programmed by changing the LED array pattern accordingly. Typically, we capture an additional two images in order to compute both the Left-Right and Top-Bottom phase derivative results representing both orthogonal directions. DPC images are qualitatively similar to Differential Interference Contrast (DIC); however, the latter is not a quantitative method. To obtain quantitative phase from DPC images, we solve the inverse problem [23, 29] using a simple deconvolution in Fourier space, as shown in Fig 3.

**Fig 4:**
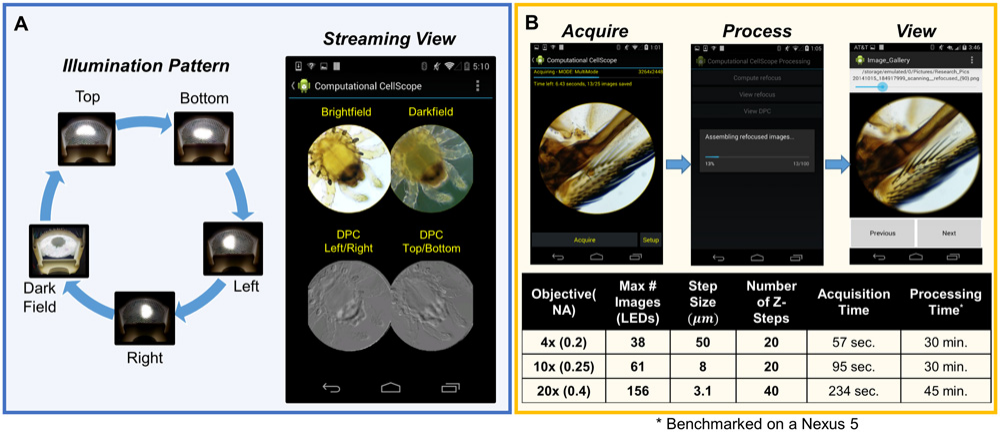
Android Application Workflow. **A.** Schematic of streaming multi-contrast LED patterns. Here we vary the LED pattern in time and acquire and process images on the smartphone, producing a streaming multi-contrast display of a sample without any further post-processing. The user can touch any image to zoom in and stream an individual image. Total cycle time is 2.3 seconds. **B.** Overview of workflow for digital refocusing mode. Table shows example processing and acquisition times for a typical dataset reconstruction. Axial Resolution is determined by the range of illumination angles sampled (defined by the objective NA). The number of z-steps were chosen such that refocus blur does not exceed 20 pixels. Processing and acquisition time can be reduced by selecting fewer refocus planes or by sparsely sampling LEDs, trading axial resolution for faster acquisition time.

Thus, by acquiring two (or four) half-brightfield images and a single darkfield image for each time point, we can synthesize brightfield, darkfield, and phase contrast modes in near real-time. Users have the option of saving and post-processing time-multiplexed frames or viewing a live multi-contrast display of the sample, though display speed is significantly faster in the latter case. We developed an application to stream these four contrast modes size-by-side while updating each frame sequentially as the illumination pattern cycles through the different patterns (Fig 4A). The user may touch any of the four images for a live full-screen display of that contrast mode only, and the illumination pattern cycle will update to reflect this.

Some image results for each of the contrast modes are shown in Fig 3, using different objective magnifications and samples. For comparison, we show the same samples imaged in a commercial inverted microscope with traditional hardware. Darkfield was obtained by using a Ph3 condenser aperture in combination with objectives having NA smaller than the sine of the half-angle of the Ph3 annulus inner diameter. Since DPC is not currently commercially available, we instead compare our DPC phase contrast images to (similar-appearing) DIC. Both provide images whose contrast is related to the first derivative of phase along a single direction; however, DIC mixes absorption and birefringence information with phase, so that dark features in the image may result from either absorption of the sample or phase contrast interferences. In the DPC images, on the other hand, the image is related purely to the sample phase distribution (see Fig 3), which can be inverted to reveal quantitative phase, as shown in the bottom row. Provided in a portable package, these multi-contrast video and streaming methods have the potential to allow clinicians to view a sample with three separate contrast methods at once, enhancing the information available for diagnosis and disease discrimination.

#### Digital Refocusing

For thick samples, our system can capture a different sequence of images in order to recover 3D images and enable digital refocusing. In this case, we sequentially capture images for each of the LEDs in the brightfield region. The resulting dataset is similar to limited angle tomography with many angles, which provides depth sectioning from angular information [35]. For simpler processing more amenable to mobile phone programming, we use a lightfield approach here [19, 28]. This involves a simple shift-and-add algorithm to digitally refocus the image to different axial (*z*) planes. We calculate the digitally refocused intensity image at a distance Δ*z* away from the physical focus plane as:

$$\begin{array}{c} I^{\Delta z}=\sum_{\text{all}\;\text{brightfield}\;\text{LEDs}}I_{i}\left(x+\Delta z\tan\theta_{x},y+\Delta z\tan\theta_{y}\right), \end{array}$$ (2)

where *I*_*i*_ denotes the intensity image for the *i* ^th^ LED, shifted according to its angle of illumination at the sample (*θ*_*x*_, *θ*_*y*_) and the desired refocus distance Δ*z*.

The number of individual LEDs making up the brightfield region roughly determines the number of depth planes that can be accurately reconstructed, and the range of illumination angles determines the axial resolution of the 3D result. Conveniently, the illumination angles may be flexibly sub-sampled in order to trade off acquisition time for quality of result. Since a separate image is taken for each illumination angle, both acquisition and processing time are a function of the numerical aperture of the objective, as illustrated in Fig 4. Acquisition speed was primarily limited by the time required to save an image to the smartphones flash memory at full resolution (8 Megapixels on the Nexus 4). This is important because data acquisition remains fast, while processing can occur in the background. Using the same dataset, we can also calculate 3D phase contrast images by digitally refocusing the two halves of the brightfield region separately [20]. It is expected that this mode of imaging intensity or phase in 3D with no moving parts will give better diagnostic information for thick samples. Alternatively, it could be used for correcting misfocus, obviating the need for automatic axial translation or automated focus adjustment in long time-lapse studies.

**Fig 5:**
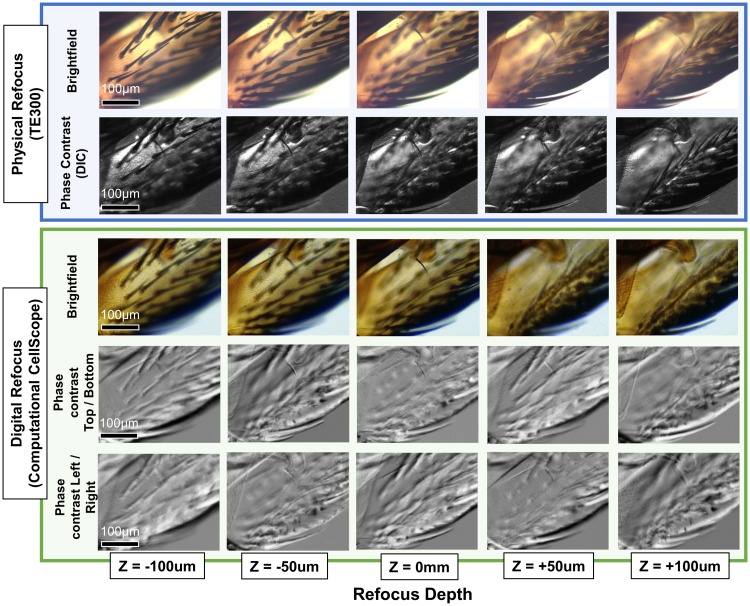
Digital refocusing on the Computational CellScope. Comparison of digital refocusing to physical refocusing on a commercial microscope (Nikon TE300) of a house fly wing sample (AmScope PS200) with a 10× objective. Digitally refocused phase contrast images are also computed for both vertical and horizontal phase derivatives at different focus depths.

Results are shown in Fig 5 for digitally refocused images as compared to physically refocused images on an inverted microscope (Nikon TE300), both with a 10× objective (0.25 NA). The phase contrast images show the first derivative of phase along both the vertical and horizontal directions, calculated from the same dataset using only the green color channel. The algorithm successfully refocused features across 400*μ*m depth of field, limited by object thickness. Our refocusing achieves an axial resolution of approximately 5*μ*m within ±50*μ*m of physical focus position, but degrades approximately linearly with increasing refocus distance [20]. Processing time is approximately 1.5 minutes per depth slice for a 10× objective.

## Materials and Methods

### Domed LED Illuminator

The illuminator consists of 4 major components: a hemispherical dome frame for mounting the LEDs, the LEDs themselves, controller circuit boards and the sample stage. The dome mounting structure is a rigid hemisphere designed to constrain the individual LEDs within an array of computationally positioned bores, aligning the LED with the radius vector to the sample center. This hemisphere was designed with a 60mm radius in order to provide maximum intensity at the sample, given our desired number of LEDs and a minimum distance between neighboring LEDs. The part was 3D printed using a SLA printer (InterPro Models) to achieve the necessary 100*μ*m printing resolution for accurate LED positioning. The LED angular positions were computed algorithmically to ensure uniform spacing across the dome, constrained by a minimum 150*μ*m distance between bores for mechanical rigidity and a maximum angular separation of 3.85 degrees allowing for sufficient coherence area at the sample. This angular spacing means that 38 LEDs make up the brightfield region for our smallest NA objective (4×), with even more for larger NA objectives, ensuring high quality digital refocusing results across a large range of depth slices for all objectives. The 3mm through-hole, white LEDs (Mouser 593-VAOL-3LWY4) were press fit into the dome and a rigid lateral constraint was provided by acrylic retaining inserts behind each individual LED. 508 of these LEDs were soldered directly to controller boards arranged above the array, with insulated leads to prevent electrical shorting.

Accounting for mechanical tolerances of the 3D printed dome and the LED epoxy lenses, manufacturing tolerances suggest that the maximum angular pointing error will be no greater than ±4.8 degrees. This corresponds to a maximum intensity attenuation of only 1.2% due to assembly variation and tolerances across all illumination angles. Our illumination is also quite uniform across the field of view. The maximum field of view of our optical system has a radius of 1.25mm, set by the eyepiece field-stop diameter of 10mm and assuming a 4× objective. Given the 60mm radius of curvature of our dome, this corresponds to illumination variation due to mechanical tolerances being less than 1% across the field of view for each single LED illumination. While this result is quite good, the spread of intensities between different LEDs is significantly larger (see Fig 2G), as a result of combined mechanical, electrical, and parts tolerances. Conveniently, a one-time calibration sweep of illumination angles, taken with no sample present, is sufficient to allow computational removal of this variation for all practical purposes.

The device used nine identical printed circuit boards placed in a fanned arrangement above the dome, each containing four LED controller chips (Texas Instruments TLC5926) serving up to 64 LEDs. These were controlled by a single Arduino Micro micro-controller, which calculates the appropriate bit pattern based on serial commands from an included Bluetooth transceiver. The array is fully addressable through a standard Bluetooth serial link; no wired connection to the phone is needed, although a powered USB connection is provided to charge the phones battery as well, for convenience. We operate the serial output at 115K baud and note that we can update the entire pattern with approximately 100ms latency, although we predefine some of the more complex LED illumination patterns and store them in the Arduino flash memory to further improve acquisition time. Thus our final acquisition time is primarily limited by the smartphone camera rather than the LED array control, so could be improved significantly with future smartphone releases.

The dome’s power control board is tolerant of voltages between 7 and 20 VDC to allow compatibility with a large range of power sources, including a standard 12V automotive battery and a 100W wall-plug variable output power supply, as well as many commercially available portable power supplies for consumer electronics. During regular usage, the device requires no more than 2A of current, though it could potentially draw up to 4.8A of current when all LEDs are illuminated. This is not a typical use case, however, since simultaneous illumination inside and outside the objective NA amounts to an undesirable mixing of darkfield and brightfield contrast. Noting that for 4×, 10×, and 20× objective configurations there are more darkfield than brightfield LEDs, to reduce power consumption we perform darkfield illumination by default using an annulus with a width equivalent to 0.15NA rather than using all of the darkfield LEDs. This moderately reduces the contrast and resolution of darkfield images but significantly reduces power use during the darkfield illumination cycle. We note that the device can operate indefinitely without overheating issues for both multi-contrast and digital refocusing.

### Acquisition Software

It has previously been shown that using a smartphone as a microscope poses unique challenges intrinsic to the phone software [14]. Smartphone cameras may only allow minimal quantitative control over standard imaging parameters (e.g. focus, exposure, gain), opting for opaque automatic algorithms that simplify the user experience. To circumvent these restrictions, we wrote a custom Android application that attempts to achieve the optimum imaging characteristics with our coded illumination configuration. In addition to a global tap-to-auto-focus capability, specific settings for each acquisition mode are detailed where appropriate in the following discussion. The software application also initiates and handles the Bluetooth connection to the domed array, enabling synchronized acquisition and array control through the standard Android API. Array control is thus transparent to the end-user, requiring them to simply pair the phone with the illuminator and press a connect button to initiate a Bluetooth connection. Our application was developed specifically for the Android platform, and will be compatible with any phone running Android OS version 4.0 or later. However, our algorithms were developed using the OpenCV Library, which is cross-platform for iOS (Apple, Inc.) and other operating systems. Thus most of our application code is portable to other smartphone platforms with moderate development effort. A screenshot of the acquisition and processing are shown in Fig 4. The user may choose to collect and synthesize any or all of darkfield, refocused brightfield and DPC images.

In our application, images were acquired using the standard Android API, which does not provide an interface to set explicit exposure times in lieu of auto-exposure with predefined exposure offset values that are only effective while auto-exposure is active. To circumvent this issue, we included a short pre-illumination sequence before each dataset acquisition to lock exposure at the appropriate value. Additionally, to account for the asymmetric LED packing of our LED array, we choose equal numbers of LEDs for each half-circle used to form DPC images, since DPC requires symmetric and equal illumination. Finally, we incur significant latency between the camera shutter and the availability of the frame to our application within the API, due to post-processing algorithms integral to the phone and performed in the background (e.g. white balance and demosaicing). This severely limits our acquisition speeds, which will likely be improved in newer versions of Android that allow finer camera control through the API. Apple iOS offers a different camera API that may also offer improvements in acquisition speed.

### Processing

Data post-processing was performed in a standalone Android app, where image stacks were loaded and processed on the phone. We employ a number of functions of the OpenCV Imaging library for Android to perform most of our computation. Individual DPC images are computed in less than a second, as demonstrated in our multi-contrast view mode. Digitally refocusing an image into 21 depth planes (±100 *μ*m range with 10 *μ*m sectioning) requires approximately 30 minutes of processing time, but the resulting 3D image stack can be interacted with in real-time; all other computational imaging results are much faster (~ 0.43 frames/sec). The long processing time is attributed to frequent loading and saving to the smartphones internal storage. We note that significant improvement in processing speeds for all of our algorithms is possible through implementation of our algorithms using the Android NDK, and is also expected as phone computational power increases with each product generation. These performance metrics were calculated on a Nexus 5 smartphone (LG Electronics) and may vary on other devices.

### Conclusions and Future work

We have built a programmable domed LED array attachment and software for the CellScope mobile microscopy platform, enabling computational illumination techniques in a portable and affordable package. We demonstrated that this device allows multi-contrast imaging in near real-time, darkfield imaging without the cost or complexity of mechanical inserts, and phase imaging methods for label-free imaging of transparent cells and microorganisms in the field. Since our LED array is programmable, we are able to switch between contrast modes in real-time, simply by changing the illumination patterning. We currently stream at a frame rate of approximately 0.43 frames per second. Additionally, we use lightfield methods to enable digital refocusing of samples and have shown that this device is capable of refocusing samples across a 400*μ*m range (at NA 0.25) with axial resolution of a few microns.

Future work will utilize the same hardware but add new imaging modes to our Computational CellScope device for quantitative 3D phase and super-resolution imaging. 3D imaging with tomographic inversions of both phase and amplitude information should be possible using similar datasets and more sophisticated algorithms [29], if they can be adapted to mobile phone post-processing. One exciting potential extension of the device is to implement Fourier Ptychography [36–38] or other super-resolution methods [1, 10] for achieving very wide field of view in conjunction with resolution well beyond the diffraction limit of the objective. These methods can also be combined with light field refocusing to obtain 3D stacks of both phase and intensity with large space-bandwidth product [29]. Our dome has been designed with these further applications in mind, so that these new capabilities can be added to our Computational CellScope platform as entirely software-side improvements. Thus, they may be deployed to existing devices via software updates over a cellular data network.
